# The Application of Design Thinking in Developing a Deep Learning Algorithm for Hip Fracture Detection

**DOI:** 10.3390/bioengineering10060735

**Published:** 2023-06-19

**Authors:** Chun-Hsiang Ouyang, Chih-Chi Chen, Yu-San Tee, Wei-Cheng Lin, Ling-Wei Kuo, Chien-An Liao, Chi-Tung Cheng, Chien-Hung Liao

**Affiliations:** 1 Department of Trauma and Emergency Surgery, Chang Gung Memorial Hospital, Chang Gung University, Linkou, Taoyuan 33328, Taiwan; detv090@gmail.com (C.-H.O.); b9402011@cgmh.org.tw (Y.-S.T.); m0102@cgmh.org.tw (L.-W.K.); m8407@cgmh.org.tw (C.-A.L.); surgymet@gmail.com (C.-H.L.); 2 Department of Rehabilitation and Physical Medicine, Chang Gung Memorial Hospital, Chang Gung University, Linkou, Taoyuan 33328, Taiwan; claudia5477@gmail.com; 3 Department of Electrical Engineering, Chang Gung University, Taoyuan 33327, Taiwan; weiclin@mail.cgu.edu.tw

**Keywords:** design thinking, artificial intelligence, deep learning, trauma, hip fracture

## Abstract

(1) Background: Design thinking is a problem-solving approach that has been applied in various sectors, including healthcare and medical education. While deep learning (DL) algorithms can assist in clinical practice, integrating them into clinical scenarios can be challenging. This study aimed to use design thinking steps to develop a DL algorithm that accelerates deployment in clinical practice and improves its performance to meet clinical requirements. (2) Methods: We applied the design thinking process to interview clinical doctors and gain insights to develop and modify the DL algorithm to meet clinical scenarios. We also compared the DL performance of the algorithm before and after the integration of design thinking. (3) Results: After empathizing with clinical doctors and defining their needs, we identified the unmet need of five trauma surgeons as “how to reduce the misdiagnosis of femoral fracture by pelvic plain film (PXR) at initial emergency visiting”. We collected 4235 PXRs from our hospital, of which 2146 had a hip fracture (51%) from 2008 to 2016. We developed hip fracture DL detection models based on the Xception convolutional neural network by using these images. By incorporating design thinking, we improved the diagnostic accuracy from 0.91 (0.84–0.96) to 0.95 (0.93–0.97), the sensitivity from 0.97 (0.89–1.00) to 0.97 (0.94–0.99), and the specificity from 0.84 (0.71–0.93) to 0.93(0.990–0.97). (4) Conclusions: In summary, this study demonstrates that design thinking can ensure that DL solutions developed for trauma care are user-centered and meet the needs of patients and healthcare providers.

## 1. Introduction

Deep Learning (DL) is a rapidly evolving subcategory of machine learning and is proving to be especially valuable in the healthcare sector [1,2,3,4]. DL has revolutionized medical image analysis and has the potential to transform healthcare delivery [5,6,7,8]. Previously, medical image analysis relied heavily on manual interpretation by radiologists and physicians, which was often time-consuming and prone to human error. However, DL algorithms have demonstrated remarkable accuracy in analyzing medical images. They have been successful in performing several classification tasks, such as diagnosing lesions, analyzing images, and classifying radiography abnormalities, comparable to or even exceeding those of human experts [9,10,11,12,13].

The integration of DL algorithms in trauma care has the potential to revolutionize the field by fostering the development of innovative solutions [14,15]. Despite its promise, the application of DL algorithms in trauma care is still in its nascent stages when compared to other sectors [16,17,18]. In trauma management, time is of the essence, and a timely and accurate diagnosis, coupled with appropriate management, can make a significant difference in the final prognosis. While physician experience is essential for providing high-quality clinical trauma care and treatment [19,20], information from various imaging modalities is also critical [21,22,23]. The goal of incorporating DL in trauma care is to hasten the diagnostic process, streamline therapeutic decision-making, and ultimately improve patient outcomes [24,25]. Therefore, it is crucial to develop an iterative DL algorithm that can be effectively implemented in clinical practice.

Design thinking is a problem-solving approach that prioritizes empathy, prototyping, and collaboration to generate innovative solutions [26,27]. It is a human-centered approach that places the needs, wants, and preferences of end-users at the center of the design process [28]. Several authors and educators have applied design thinking to medical education [29,30,31,32] and medical device development [33,34]. The design thinking process typically involves five steps: empathy, define, ideate, prototype, and test. The empathy stage involves understanding the needs and preferences of the end-users, including clinical physicians and patients. The define stage involves defining the problem and identifying the criteria needed for success. The ideate stage involves brainstorming potential solutions to the problem. The prototype stage involves creating a tangible representation of the solution. Finally, the test stage involves testing the prototype with end-users and gathering feedback to improve the solution.

Although design thinking has shown great breakthroughs in other areas [35,36,37,38,39], there is no previous experience in developing DL algorithms for trauma care. The integration of design thinking into the development of DL algorithms for trauma care has the possibility to modify clinical decision-making and improve patient outcomes. By prioritizing the needs and preferences of end-users, design thinking can lead to the creation of more effective and user-friendly tools [40].

In this study, we aim to demonstrate that incorporating design thinking into the development of DL algorithms for trauma care can help clinical physicians to create the most appropriate algorithm for a clinical scenario. Furthermore, we conducted a multicenter validation to prove the performance of the design thinking-based algorithm.

## 2. Materials and Methods

**Figure 1:**
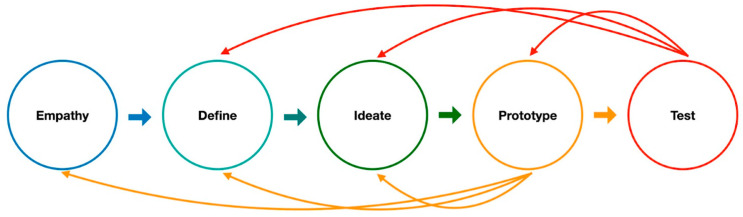
The design thinking process: the five steps include empathy, define, ideate, prototype, and test. Each step can be repeated individually, allowing for an iterative approach to problem-solving.

We utilized the design thinking process to develop a solution to solve clinical problems. Design thinking can be a useful approach for medical innovation. The process includes five steps: empathy, define, ideate, prototype, and test, with continuous iteration throughout the process [26]. The five steps of design thinking where empathy, define, ideate, prototype, and test (Figure 1).

By utilizing this approach, we were able to develop a solution to address the clinical problems faced by physicians. To ensure that our study was ethical and informed, we interviewed the clinical physicians to understand the clinical unmet need and defined the research question. All of the physician participants were well-informed and consented to participate in the study.

Furthermore, we utilized the Chang Gung Trauma Registry Program (CGTRP) in Chang Gung Memorial Hospital (CGMH), Linkou, Taiwan. Demographic data, medical data, perioperative procedures, hospital procedures, medical imaging findings, follow-up data, and information regarding complications were recorded prospectively in a computerized database. We extracted the data and images of all trauma patients treated from August 2008 to December 2017 at CGMH, which is a level I trauma center for further DL algorithm development. The Internal Review Board of CGMH approved the study with No: 202002343B0.

### 2.1. Empathize the Clinical Physicians

The first step in design thinking is to empathize with the user. In the context of surgical innovation, this means understanding the needs and challenges of both the patient and the surgical team. We interviewed the physicians before developing the algorithm and labeling the data. The trauma surgeons who work in the trauma bay were interviewed for clinical challenge search and identification. We conducted the interviews to collect any issues about clinical difficulty and to explore the problem.

### 2.2. Defining the Clinical Unmet Needs and Ideating the Solution for the Clinical Unmet Needs

The research team initiates the process by gathering insights and defining the problem or opportunity. This step will guide the rest of the process of clinical issues and unmet needs into one dominant question that is used to further develop the algorithms. Through brainstorming sessions involving a multidisciplinary team, a broad range of ideas and potential solutions to the problem or opportunity were generated. This involves adjusting the process and concept to integrate the clinical problems with the technological solutions. In the present study, we would like to define the unmet needs and generate ideas for the application of a proper deep learning algorithm to assist clinical physicians in addressing their specific challenges.

### 2.3. Prototyping the DL Algorithm

The next step is to create a prototype of the potential solution. For this step, we developed a DL algorithm based on the dataset of CGTRP. The PXR was collected from patients registered from May 2008 to December 2016. The demographic and trauma-related data including age, gender, date of injury, mechanism of injury, Abbreviated Injury Scale (AIS), final diagnosis, and outcome, were recorded. We extracted the final diagnosis of hospitalization, operative finding, and the anteroposterior PXR of the patient was acquired from the picture archiving and communication system (PACS) repository.

**Figure 2:**
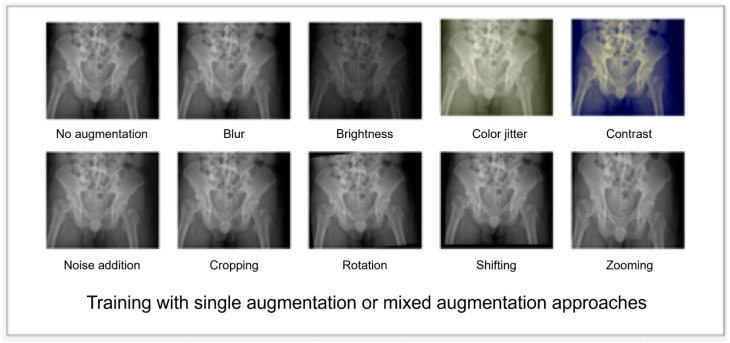
The augmentation methods used for training this deep learning algorithm.

The architecture of a DL algorithm in this study is based on Xception [41], a convolutional neural network (CNN). The CNN architecture combines depth-wise separable convolutions with residual connections to achieve high accuracy with fewer parameters and reduced computational requirements. The architecture of Xception is based on the idea of using depth-wise separable convolutions, which split the standard convolution operation into two separate operations: depth-wise convolution and pointwise convolution. The depth-wise convolution applies a single convolutional filter to each input channel independently, while the pointwise convolution applies 1 × 1 convolutions to combine the output channels of the depth-wise convolution. This separation reduces the computational cost of convolutions by factorizing the standard convolution operation. The images were resized into 512 × 512 pixels on the maximum dimension with zero paddings. The input of the model is the whole image, and the output of the model is a binary classification result representing either hip fracture or no hip fracture. We used 10 image augmentation methods, including blur, brightness, color jitter, contrast adjustment, noise addition, cropping, rotation, shifting, and zooming during the training process (Figure 2).

The images after augmentation methods applied were reviewed to check whether the visual features of positive class images can still be recognized. During the training process, the augmented images were randomly added. To test the effect of each image augmentation method, we strategically utilized a diverse set of 10 distinct augmentation methods, each applied individually, to train our model. This approach allowed us to explore the unique impact of each augmentation technique on the performance of the model. Building upon this initial stage, we then proceeded to combine all 10 augmentation methods together, employing a mixed model of training. This subsequent training phase aimed to comprehensively assess and analyze the variations in model performance resulting from the combined use of these diverse augmentation techniques. The models were initialized with the ImageNet pre-trained weight. During the training process, each model was trained with the Adam optimizer using an initial learning rate of 10^−3^, a batch size of 8, and stopped at 70 epochs. We used Gradient-weighted Class Activation Mapping (Grad-CAM) [42] to visualize the portion of the image that the model focused on to give the prediction. The data of the development dataset from hospital A was divided to 80%, 10%, and 10% as the train, validation, and test dataset, respectively.

We utilized TensorFlow 2.2.0 with Keras 2.3.1, running on Python 3.8.2, to build the structure of the DL algorithm on the Ubuntu 18.04 operating system. The whole training process was run on a GeForce^®^ GTX 1080 Ti GPU. Based on our current hardware configuration, we conducted 70 epochs of training for each model, with each epoch requiring approximately 4 min to complete. Consequently, the entire training process for a single model lasted approximately 280 min.

### 2.4. Testing, Validating, and Remodeling the Algorithm

The final step is to test the prototype with the testing dataset from CGMH. The test set consisted of 100 PXRs including 25 femoral neck fractures, 25 intertrochanteric fractures, and 50 without fracture. We evaluated the performance of the algorithm and incorporated GradCAM visualization to aid user recognition. Following the iterative design-thinking cycle, we carefully reviewed the results and conducted user interviews to gather insights regarding their perception of the algorithm. Specifically, we sought feedback on the performance of the algorithm, the effectiveness of augmentation methods in accounting for variations in clinical environments, and the accuracy of heatmap localization generated by each developed model.

Based on the feedback received, we made enhancements to the algorithm. Instead of solely relying on zoom, horizontal flip, vertical flip, and rotation as augmentation methods in the original version, we introduced multiple additional augmentation techniques to increase the generalizability of the model. Furthermore, we refined the presentation of the algorithm to better cater to user needs and address problem areas identified during the review process.

### 2.5. Statistical Analysis and Software

The analysis of continuous variables involved using the Kruskal–Wallis rank-sum test for comparison. For categorical variables, the Chi-square test and Fisher’s exact test were used for comparison. 

The performance of the model was evaluated with the receiver operating characteristic (ROC) curve. The area under the ROC curve (AUC) was used for evaluation of the performance of the model.

## 3. Results

### 3.1. Finding Empathy and Definition

Five trauma surgeons were interviewed to assess their approach to clinical challenges and to identify any unmet needs in handling emergency trauma cases at our hospital. A total of eight unmet needs were identified by the surgeons. These include: (1) detecting a life-threatening hemorrhage that required immediate intervention; (2) predicting critical head injuries that require surgery or intensive care; (3) predicting delayed trauma presentation with secondary complications; (4) determining the need to apply anticoagulant agents and intervention to prevent trauma-induced coagulopathy; (5) detecting subtle fractures in essential x-ray images, such as chest plain films and pelvic plain radiographs (PXR); (6) determining the necessity of surgery in mild blunt or penetrating trauma patients (7) predicting infections or sepsis in patients with multiple trauma; and (8) diagnosing spinal cord injuries that require urgent diagnosis and management. We selected the most frequently reported issue among the surgeons as our primary focus for the study. This issue was detecting subtle fractures in essential x-ray images. Our research team initiated internal discussions and brainstorming sessions, with specific emphasis on the undiagnosed subtle fractures that could result in dismal sequelae such as avascular necrosis, using the principles of design thinking. Finally, the clinical unmet need was defined as “how to reduce the misdiagnosis of femoral fracture by PXR at initial emergency visiting”. To address this unmet need, we developed a visualization-based deep learning algorithm to help clinical physicians identify the presence of possible fractures in PXR in the emergency department.

### 3.2. Prototyping—Testing Cycle to Improve the Performance of the Algorithms

**Figure 3:**
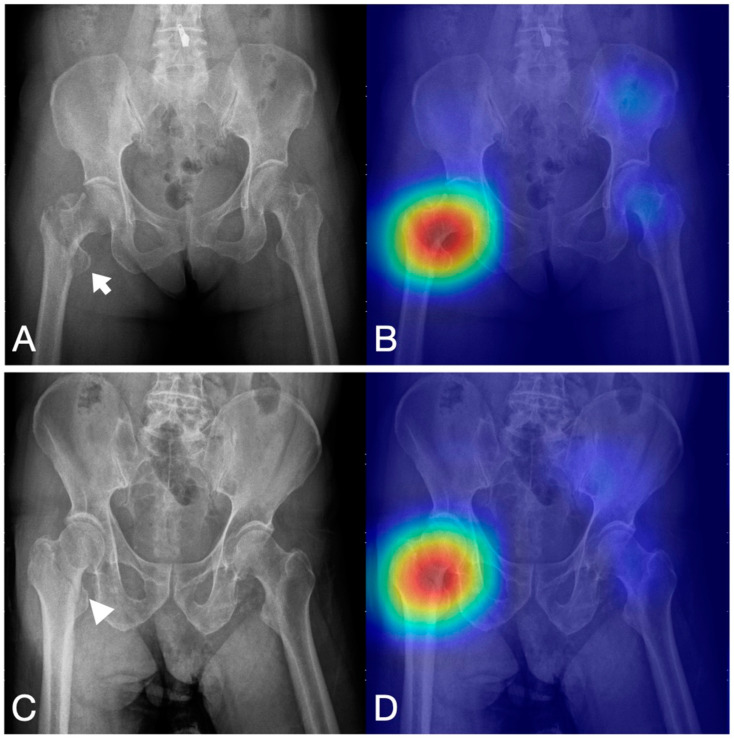
The clinical presentation of the deep learning algorithm for hip fracture detection. (**A**) The plain pelvic X-ray shows a displaced right intertrochanteric fracture, with the fracture line indicated by an arrow. (**B**) The heatmap highlights the fracture location, providing clinical doctors with a reference for review. (**C**) The plain pelvic X-ray shows an occult right intertrochanteric fracture without displacement. The arrowhead indicates the fracture line. (**D**) The heatmap again indicates the fracture location for review by clinical doctors.

We collected 4235 anteroposterior PXRs from our hospital, of which 2146 had a hip fracture (51%) from 2008 to 2016. Hip fracture DL detection models based on the Xception CNN were developed using these images. We trained a model without any image augmentation method as a baseline. The developing details were listed in our previous study [43]. In the last layer, we applied Grad-CAM to highlight the lesion predicted by the DL algorithm and explain the process of the algorithm. On the visualization heatmap, all the models highlighted the fractured hip on the PXR image, therefore, we can use the image to assist the clinical doctors to detect the possible area (Figure 3).

After the models developed, the models were directly applied to the emergency department for evaluating the clinical performance of hip fracture detection. The initial results are shown in Table 1. The accuracy, sensitivity, specificity, false-negative rate, and F1 score of the model were 91% (*n* = 100; 95% CI, 84–96%), 97% (95% CI, 89–100%), 84% (95% CI, 71–93%), 2% (95% CI, 0.3–17%), and 0.916 (95% CI, 0.845–0.956), respectively. The details of these aspects were comprehensively described in a previous study [43]. However, unlike previous studies, we implemented this model in a clinical setting and found several limitations in its application. Furthermore, we reevaluated the clinical feedback and engaged in a process of rethinking to make the necessary modifications to the algorithm to suit its deployment status. Using the feedback from clinical and background calculation, we remodeled the algorithm by using individual image augmentation methods and a model using a mixed method. In addition, the clinical requirement is as follows: “ensure that all the patients discharged from the hospital are free from any fractures”. Therefore, we refine the issue as “increase the sensitivity and reduce the false negative rate”. The cut-off value of the algorithm was adjusted. Furthermore, we refined the presentation from the initial yes/no fracture shift to the visualized presentation, which increased the acceptance of clinical users. Initially, we offered a yes/no fracture presentation to the clinical doctors who did not prefer this suggestion and gave our team their opinion to modify it. After discussion, the algorithm was designed to analyze the X-ray images and provide visual cues to the physician for any possible fracture locations. The physician could then confirm the diagnosis with further investigation or imaging. By these modifications, the performance of the post-design thinking model on the dataset was shown in Table 1. Some of the models trained with augmentation methods, including blur, contrast adjustment, image shifting, and mixed augmentation, performed significantly better than the baseline.

**Figure 4:**
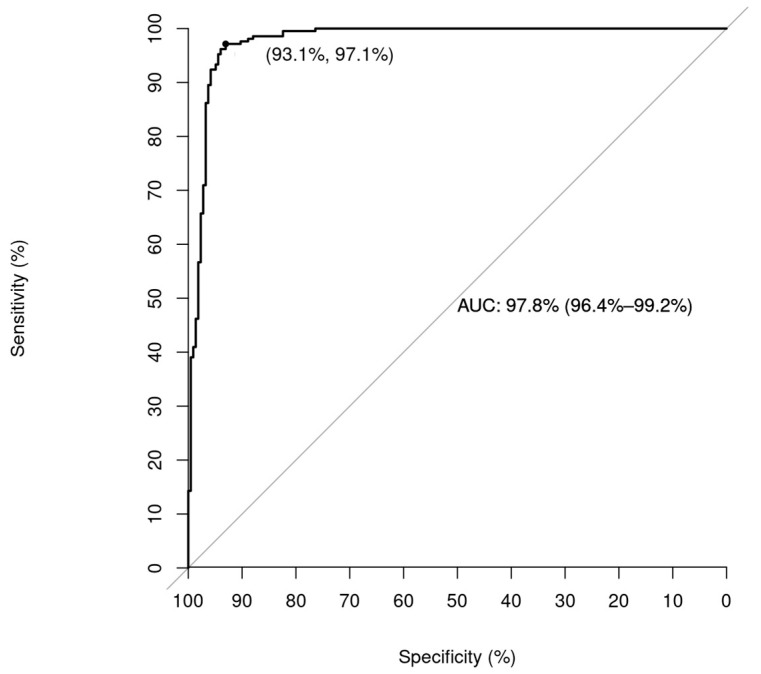
The Receiver Operating Characteristic (ROC) curve of the Post-DT model is presented. The point on the ROC curve signifies the optimal cut-off point based on Youden’s index and also represents the sensitivity and specificity of the model.

The Receiver Operating Characteristic (ROC) curve of the Post-DT model is presented as Figure 4. We can identify the area under the curve as 0.97.

## 4. Discussion

In this study, we presented a project based on Design Thinking to develop DL algorithms that meet the needs of end-users. By identifying their needs quickly and testing the prototypes, we were able to make modifications and deploy the algorithms rapidly. By incorporating feedback from clinical doctors, we were able to improve the diagnostic accuracy from 91% to 95%, the sensitivity from 97% to 97%, and the specificity from 84% to 93%. Trauma care is time-sensitive, and the use of deep learning algorithms can significantly improve the diagnostic rate and time. With the support of these projects, clinicians can make more accurate diagnoses and provide timely treatment to patients.

The management of trauma patients is a complex process, and time is of the essence. Triage plays a crucial role in differentiating patients into appropriate dispositions, and according to advanced trauma life support, it is the first step before the primary survey in managing severely wounded patients. The PXR is a low-cost tool that is essential for detecting possible fractures in trauma patients [44]. With the support of innovative information technology, we have developed AI algorithms capable of accurately classifying trauma patients within a constrained timeframe. Deep learning technology has been applied to the medical sector for years, and previous works have focused on fracture detection by plain films with excellent performance [6,15,45,46]. Meanwhile, continuous improvement and follow-up results are necessary to ensure optimal performance. In this study, we provide a follow-up and improved performance results of the DL algorithm in fracture detection systems. Once a deep learning algorithm is developed, continuous input of the labeled data can improve its performance [47]. By incorporating increasing data, the algorithm can be trained to recognize new patterns and improve its accuracy. Additionally, clinical feedback is essential for improving and modifying the algorithm to fit clinical needs. This feedback loop is critical for the ongoing development and deployment of deep learning algorithms in clinical practice [48,49].

The continuous improvement of our algorithm is heavily influenced by the insights and expertise of physicians, particularly when it comes to adjusting augmentation methods. Augmentation methods play a crucial role in generating images that serve as valuable training material, enabling us to enhance the generalizability of the algorithm. Physicians provide essential guidance by describing the potential changes that images may undergo in clinical environments, such as blurring and shifting caused by patient movement, as well as variations in brightness and contrast across different machines. These inputs from physicians are invaluable as they help us create augmentation strategies that better reflect real-world scenarios. In the early stages, our algorithm relied solely on traditional augmentation methods, which inevitably limited its generalizability. To address this constraint, we embarked on an iterative journey, systematically integrating various augmentation techniques. Based on the recommendations of the physicians, we constructed model prototypes to experiment with and refine the algorithm. Each iteration allowed us to evaluate the effectiveness of various augmentation methods in enhancing the performance. Through this iterative process, we progressively incorporated a diverse range of augmentation methods. These included techniques for image cropping, rotation, shifting, and zooming, as well as adjustments to blur, brightness, color jitter, contrast adjustment, and noise addition. By integrating multiple augmentation strategies, we aimed to expose the algorithm to a wider range of data variations, thereby enhancing its ability to generalize to unseen scenarios. Once all the augmentation methods were integrated, we observed a significant improvement in performance. The algorithm demonstrated enhanced robustness and adaptability, showcasing its ability to handle various clinical environments and imaging conditions effectively.

The deployment of novel DL algorithms in clinical practice has the potential to revolutionize the field of medicine. However, there are multiple factors that must be considered to ensure successful implementation in real-world settings. Clinical doctors may lack confidence in the new tool, especially if the workings of the algorithm are not explained clearly [50,51,52]. The heavy computational power and cost of hardware are additional considerations that must be taken into account. Furthermore, most deep learning algorithms only solve one problem separately, which may not be compatible with the clinical workflow and the expectations of frontline physicians [6]. As a result, the deployment of deep learning algorithms in medical scenarios is still in its initial phase. To overcome these challenges, following the design thinking process can help clinical physicians develop deep learning algorithms that are tailored to the specific needs of trauma care [53]. Initially used for business development, the power of design thinking has been recognized for its applicability in various sectors, including the medical field. The Stanford Byers Center for Biodesign has applied design thinking to develop a range of medical innovations [54,55], including surgical tools and devices [56,57]. They have developed a specific process for medical innovation that involves identifying clinical needs, brainstorming potential solutions, creating prototypes, and testing the prototypes with users to refine the design [58]. Some educators use design thinking to arrange and allocate the resources and the coaching system for medical students and young medical residents with great performance [59,60]. In this study, we used design thinking to arrange the process of DL algorithm development. Involving end-users in the design process is critical to ensure that deep learning algorithms are developed with their needs in mind, leading to increased adoption and usability. By involving frontline physicians in the design process, clinical feedback can be gathered, and the algorithm can be modified to fit clinical needs. This approach can lead to a more effective and efficient deployment of deep learning algorithms in clinical practice. Continuous improvement and follow-up results are necessary to optimize the performance of the algorithms, which is compatible with the core value of design thinking. Rapid iteration of the design thinking cycle and improving the performance of algorithms can enhance the confidence of clinical doctors in the project.

### Limitations

While this study demonstrated the efficacy of the design thinking-based deep learning algorithm and its potential for clinical adoption, there were several limitations that should be acknowledged. First, although we attempted to minimize selection bias by randomizing the selection of images, it cannot be completely eliminated due to the nature of the dataset composition. Future studies with larger and more diverse datasets may be required to address this limitation. Second, because the feedback from clinical doctors was an essential part of the study, it was not possible to conduct a double-blind study. This may have introduced bias into the feedback process, potentially affecting the performance of the algorithm. However, we made efforts to minimize this by using a standardized survey form and ensuring that the clinical doctors providing feedback were not involved in the development of the algorithm. Third, as this was a single-center study, the generalizability of the findings may be limited. Further institutional collaborations to expand the data number and improve labeling may be necessary to develop the algorithm according to the global user requirements. This can help ensure that the algorithm is more representative of a broader patient population and healthcare settings, leading to improved performance and more widespread adoption.

## 5. Conclusions

Design thinking can be used to ensure that the DL solutions developed for trauma care are user-centered and address the needs of patients and healthcare providers. By following the design thinking process, we can gather insights into the needs of users, ideate potential solutions, create prototypes, and test the solutions with users to refine the design. By prioritizing the needs, and preferences of physicians, we can develop DL algorithms that are tailored to the specific needs of trauma care. This can lead to more accurate diagnoses, more efficient treatment, and improved patient outcomes, ultimately contributing to the advancement of trauma care.

## Figures and Tables

**Table 1:** The difference in performance before and after the introduction of design thinking for the deep algorithm.

	Pre DT Model (95%CI)	Post DT Model (95%CI)
Accuracy	0.91 (0.84–0.96)	0.95 (0.93–0.97)
Sensitivity	0.97 (0.89–1.00)	0.97 (0.94–0.99)
Specificity	0.84 (0.71–0.93)	0.93 (0.90–0.97)
False negative rate	0.02 (0.003–0.17)	0.0286 (0.0095–0.0667)
F1 score	0.916 (0.845–0.956)	0.951 (0.930–0.973)

## Data Availability

The dataset is not publicly available due to restrictions imposed by the data sharing agreements with the Chang Gung Memorial Hospital Institutional Review Board (IRB). However, a partial dataset can be obtained upon reasonable request to the corresponding authors for academic purposes.
